# A Hypothesis That Glucagon-like Peptide-1 Receptor Agonists Exert Immediate and Multifaceted Effects by Activating Adenosine Monophosphate-Activate Protein Kinase (AMPK)

**DOI:** 10.3390/life15020253

**Published:** 2025-02-07

**Authors:** C. Mary Schooling, Guoyi Yang, Ghada A. Soliman, Gabriel M. Leung

**Affiliations:** 1 School of Public Health, Li Ka Shing Faculty of Medicine, The University of Hong Kong, Hong Kong, China; yanggy@connect.hku.hk (G.Y.);; 2 School of Public Health and Health Policy, City University of New York, Graduate School of Public Health and Health Policy, 55 W 125th St, New York, NY 10027, USA; ghada.soliman@sph.cuny.edu

**Keywords:** AMPK, semaglutide, non-communicable diseases, calorie restriction, unifying theory

## Abstract

Glucagon-like peptide-1 receptor agonists (GLP-1RAs) reduce bodyweight and blood glucose. Extensive evidence from randomized controlled trials has indicated that GLP-1RAs have benefits well beyond weight loss and glucose control, extending from reductions in cardiovascular mortality to reductions in prostate cancer risk. Notably, some benefits of GLP-1RAs for the cardiovascular–kidney–metabolic (CKM) system arise before weight loss occurs for reasons that are not entirely clear but are key to patient care and drug development. Here, we hypothesize that GLP-1RAs act by inducing calorie restriction and by activating adenosine monophosphate-activated protein kinase (AMPK), which not only provides an explanation for the unique effectiveness of GLP-1RAs but also indicates a common mechanism shared by effective CKM therapies, including salicylates, metformin, statins, healthy diet, and physical activity. Whether AMPK activation is obligatory for effective CKM therapies should be considered. As such, we propose a mechanism of action for GLP-1RAs and explain how it provides an overarching framework for identifying means of preventing and treating cardiovascular, kidney, metabolic and related diseases, as well as informing the complementary question as to the components of a healthy lifestyle.

## 1. Introduction

Targeting weight loss via glucagon-like peptide-1 receptor antagonists (GLP-1RAs) has been immensely successful. GLP-1RAs are providing a new way of reducing the burden of adiposity, enhancing quality of life, and preventing early mortality. Inevitably, evidence from randomized controlled trials (RCTs) concerning the effects of GLP-1RAs is still somewhat limited. Nevertheless, in a large RCT, a GLP-1RA, semaglutide, was found, somewhat unexpectedly, to reduce cardiovascular disease (CVD) events and mortality before substantial weight loss had occurred [1], suggesting that GLP-1RAs act via mechanisms beyond weight loss. In the same trial, semaglutide also reduced CVD more than expected from the change in body weight (−8.5%) [1], additionally suggesting that GLP-1RAs do not only act via weight loss. Meta-analysis of RCTs has also found that GLP-1RAs reduce the risk of conditions not necessarily wholly driven by adiposity, such as prostate cancer [2] and kidney disease [3,4]. Taken together, trial evidence suggests that GLP1-RAs act by mechanisms beyond weight loss. Here, we synthesize these findings concerning GLP-1RAs and place them within a comprehensive, explanatory framework, i.e., within a hypothesis, to provide a unifying explanation for the mechanisms driving the immediate and multifaceted effects of GLP-1RAs, as well as having relevance to other effective cardiovascular–kidney–metabolic disease therapies jointly [5] or singly.

## 2. Known Mechanisms

GLP-1RAs directly target insulin and glucagon, which results in reduced gastric emptying, reduced acid secretion, and increased satiety and thereby reduced appetite, food consumption, and calorie intake. The lower calorie intake undoubtedly drives some of the subsequent weight loss seen with the use of GLP-1RAs and consequent health benefits. Whether targeting insulin and glucagon directly explains the immediate and relatively large benefits of GLP-1RAs for CVD and all-cause mortality [1] is unclear. Evidence from RCTs suggests that insulin and sulfonylureas increasing insulin secretion has neutral effects on CVD and mortality for adults with type 2 diabetes [3]. Effects of glucagon have rarely been studied [6].

## 3. Potential Mechanism via Adenosine Monophosphate-Activated Protein Kinase (AMPK)

GLP-1RAs, as well as promoting weight loss, have also been suggested to be calorie restriction mimics [7]. Correspondingly, in an RCT, semaglutide reduced energy intake by 24% without changing resting metabolic rate [8]. Calorie restriction is a means of lengthening lifespan, within the broader conceptualization, originating with Darwin, that drivers of growth and reproduction trade off against longevity [9]. Genetic evidence of such antagonist pleiotropy in humans exists [10]. Trial evidence concerning calorie restriction in humans is limited to a two-year trial of 25% calorie restriction, which found substantial cardiometabolic benefits [11]. Natural experiments where reductions in food consumption without malnutrition (~20% calorie restriction) occurred, such as in Denmark from 1917 to 1918 and in Norway from 1941 to 1945, coincided with reductions in mortality rates [12,13].

Calorie restriction activates adenosine monophosphate-activated protein kinase (AMPK), which in turn inhibits the mechanistic target of rapamycin (mTOR) complex 1 (mTORC1) [14]. In mice, GLP-1RAs have been shown to activate AMPK [15]. AMPK was originally identified by Carlson and Kim in 1973 as a regulator of fatty acid synthesis [16]. AMPK has subsequently been found to have many functions in keeping with its central role in metabolism. AMPK continually monitors and manages energy homeostasis [16]. AMPK activation is consistent with many of the effects of GLP-1RAs, such as healthier lipids [16,17], healthier glucose metabolism [16,17], lower blood pressure [17,18], and improved kidney function [18,19], as well as the corresponding immediate reduction in CVD and mortality. Definitive investigation of GLP-1RAs’ impact on liver function is still underway (NCT04822181).

## 4. Role of AMPK Activation in Cardiovascular–Kidney–Metabolic Treatments and Other Therapies

Given its central role in metabolism, AMPK has been found to be activated by several long-standing effective cardiometabolic treatments with diverse origins, such as salicylates [16], metformin [16], statins [18], sodium–glucose cotransporter-2 (SGLT2) inhibitors [16], and finerenone [20]. AMPK is also activated by new or emerging treatments for CVD, such as bempedoic acid [16], and by some promising potential cardiometabolic therapies, such as berberine [18], cordycepin [21], and asialoglycoprotein receptor 1 (ASGR1) inhibitors [22]. As such, GLP-1RAs activating AMPK is consistent with other successful cardiometabolic drugs activating AMPK and explains the immediate effects of GLP-1RAs on CVD and mortality, as well as their relatively large effects. It is less obvious why GLP-1RAs activating AMPK reduces the risk of prostate cancer [2] and ameliorates major kidney disease events (kidney failure onset, reduction in estimated glomerular filtration rate of 50% or more, or death from kidney-related or cardiovascular causes) in people with both chronic kidney disease and type 2 diabetes [23]. Evidence from RCTs has shown that some drugs potentially acting via AMPK, such as statins [24] and metformin [25], reduce circulating testosterone. Part of the effect of statins on ischemic heart disease in men has been shown to be mediated by circulating bioavailable testosterone [26]. GLP-1RAs reduce testosterone in women [27]. Trial evidence concerning effects of GLP-1RAs on free and total testosterone in men is currently limited to one small trial in healthy young men with a normal BMI [28]. Circulating free or bioavailable testosterone is relevant to prostate cancer and has also been implicated in kidney disease [29]. As such, effects of GLP-1RAs on prostate cancer and kidney disease could be due to GLP-1RAs activating AMPK and thereby decreasing androgens.

**Figure 1:**
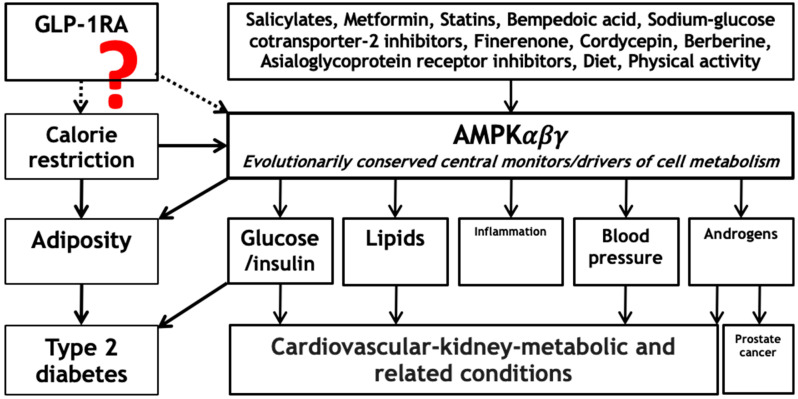
Hypothesized relations of GLP1-RAs with calorie restriction and AMPK (dotted arrows) and their established relations with cardiovascular–kidney–metabolic health and related diseases, and their major risk factors, along with other effective means of preventing or treating cardiovascular–kidney–metabolic and other diseases likely also operating via AMPK (solid arrows). Abbreviations: GLP-1RA: glucagon-like peptide-1 receptor agonist and AMPK: adenosine monophosphate-activated protein kinase.

Here, we hypothesize that GLP-1RAs act by activating AMPK, both directly [15] and via calorie restriction [30], which provides an overarching explanatory framework for the immediate and wide-range effects of GLP-1RAs (Figure 1). Several effective cardiovascular–kidney–metabolic medications activate AMPK directly (16, 18, 20). Calorie restriction promotes AMPK activation, but dietary composition also plays a role in the level of AMPK activation achieved [30], with corresponding implications for both users and non-users of GLP-1RAs. Notably, a recent investigation in female mice found that calorie and dietary restriction prolonged lifespan, beyond their effects on bodyweight and metabolic traits [31]. These findings are consistent with an upstream central driver responsive to calorie restriction, such as AMPK, affecting lifespan. Nevertheless, it is unclear whether the potential benefits of GLP-1RAs, operating via AMPK activation or otherwise, extend to cancer in general or to infectious diseases. Activating AMPK decreases cancer risk, but is thought to switch to a tumor promoter after cancer occurs [32]. Trial evidence concerning the effect of GLP1-RAs on cancer is limited, because most trials have focused on cardiometabolic outcomes. The evidence to date from trials suggests that GLP-1RAs have a neutral effect on overall cancer but increase the risk of thyroid cancer [33]. Similarly, evidence from quasi-experimental designs, such as mendelian randomization (MR), also suggests that GLP1-RAs have little effect on overall cancer risk [34]. Intriguingly, some evidence from trials suggests that GLP1-RAs may reduce death from infectious diseases [35], which requires further clarification.

Apart from GLP1-RAs acting via insulin secretion in the pancreas [36], mechanistic studies suggest that GLP-1RA activates AMPK in several different ways. GLP1-RA acting via the ventromedial hypothalamus has been shown to reduce food intake in mice and rats [37,38] due to AMPK activation [39]. GLP1-RA acts via hindbrain neurons [40], which affects AMPK activity [41]. In addition, GLP1-RAs act via vagal afferents [42], which also affect AMPK activity [43]. Correspondingly, a recent bioinformatics study suggested that GLP-1RAs might affect AMPK signaling via the circular ribonucleic acid network (RNA), specifically competitive endogenous RNA [44]. Finally, a small experimental study in pigs found that treatment with a GLP1-RA improved heart function after inducing coronary heart disease, potentially via AMPK activation [45]. Whether GLP-1RA also affects other nutrient sensors, such as mTOR and its downstream sensors, such as S6 kinase, to the same extent is less clear [46,47]. However, we are yet to establish the extent to which GLP-1RAs reduce the risk of cardiovascular-kidney-metabolic and related diseases, through direct action, their action via AMPK, their action via appetite suppression, and their interactions.

Dietary or other lifestyle factors, such as physical activity, that activate AMPK are also expected to extend life, and those that suppress AMPK may be associated with cardiovascular–kidney–metabolic diseases [30]. AMPK activation also reduces inflammation [48], as seen for semaglutide [49], meaning that inflammation might be an aspect of AMPK suppression causing cardiovascular–kidney metabolic diseases. Correspondingly, mitigating these diseases via anti-inflammatory agents has proven difficult [50].

AMPK also works in concert with its key partner mTOR. AMPK is primarily activated in metabolic tissues by nutrient-poor conditions, while mTOR is primarily activated by nutrient-rich conditions [51]. As such, AMPK is more likely the relevant pathway driving the effects of GLP1-RAs via calorie restriction, notwithstanding the key role of mTOR in chronic diseases. However, that is not to say that mTOR is an unimportant pathway or an unimportant target of intervention for preventing diseases.

## 5. Next Steps

Whether GLP-1RAs operate via calorie restriction and activating AMPK could be tested in several ways. Experimentally, AMPK activation could be tested in cell lines relating to both the drivers and the postulated effects of GLP1-RAs, such as neural, adipocyte, endothelial, and cancer cell lines, and in animal models by phosphorylation and dephosphorylation of downstream targets. It would also be helpful to clarify the role of other likely effective drivers of AMPK activation, such as specific dietary factors and types of physical activity. Similarly, it would be helpful to assess whether differing effects of different GLP-1RAs and other factors activating AMPK in tissue from the ventromedial hypothalamus, hindbrain neurons, and vagal afferents [40,41,42] are due to their differing effects on the three AMPK subunits (α, β, and γ) and the seven isoforms. Standard methods to assess activation of AMPK at the cellular level exist. In addition to experiments, drug target MR, taking advantage of existing publicly available genetic data, could be used within an established analytic framework [52], to assess in humans GLP-1RAs’ effects on cell senescence biomarkers, such as telomere length or phenotypic age. Drug target MR could also be used to assess whether GLP-1RAs act on cardiometabolic risk factors, CVD, type 2 diabetes, all-cause mortality, kidney disease, prostate cancer, and possibly liver function via AMPK. MR could also clarify whether inflammation is a mechanism driving these conditions or is a symptom of AMPK suppression. Whether GLP-1RAs reduce testosterone in men as well as in women [27] could most expediently be ascertained from an RCT of an effective GLP-1RA in the relevant target population, or possibly through re-use of stored specimens from previous RCTs. Taken together, these bench and desktop studies would not only clarify the mechanism underlying the success of GLP-1RAs, so as to optimize their use, but could also underpin and strengthen existing global advice on a healthy lifestyle. Finally, insights about the mechanism of action of GLP-1RAs may facilitate the development of new treatments for cardiovascular–kidney–metabolic and other diseases.

## 6. Concluding Remarks

Here, we hypothesize that GLP-1RAs operating mechanistically via calorie restriction and also via AMPK directly explain the immediate and long-term benefits of GLP-1RAs for the cardiovascular–kidney–metabolic system, as well as explaining the somewhat unexpected reduction in prostate cancer [2]. These mechanisms also provide a unifying explanation for the mode of operation of several effective therapies for these diseases, including salicylates, metformin, statins, SGLT inhibitors, finerenone, and bempedoic acid, as well as therapies that could possibly be more widely used after suitable testing, such as cordycepin, berberine, and ASGR1 inhibitors. Whether AMPK activation is obligatory for the effective prevention and treatment of cardiovascular, kidney, metabolic and relateddiseases across different types of interventions warrants further investigation. Finally, some of the mechanisms here, as drivers of cardiovascular disease, are likely relevant to differences in lifespan between men and women and may provide clues as to how to redress this inequity.

## Abbreviations

AMPK	adenosine monophosphate-activated protein kinase
ASGR1	asialoglycoprotein receptor 1
CKM	cardiovascular-kidney-metabolic
CVD	cardiovascular disease
GLP-1RA	glucagon-like peptide-1 receptor agonists
MR	Mendelian randomization
mTOR	mechanistic target of rapamycin
RCT	randomized controlled trial
SGLT2	sodium–glucose cotransporter-2
